# Substrate Affinity Is Not Crucial for Therapeutic L-Asparaginases: Antileukemic Activity of Novel Bacterial Enzymes

**DOI:** 10.3390/molecules29102272

**Published:** 2024-05-11

**Authors:** Anna Ściuk, Kinga Wątor, Izabela Staroń, Paulina Worsztynowicz, Kinga Pokrywka, Joanna Sliwiak, Marta Kilichowska, Kamila Pietruszewska, Zofia Mazurek, Anna Skalniak, Krzysztof Lewandowski, Mariusz Jaskolski, Joanna I. Loch, Marcin Surmiak

**Affiliations:** 1 Department of Crystal Chemistry and Crystal Physics, Faculty of Chemistry, Jagiellonian University, Gronostajowa 2, 30-387 Krakow, Poland; anna.sciuk@doctoral.uj.edu.pl (A.Ś.); marta.kilichowska@doctoral.uj.edu.pl (M.K.); 2 Doctoral School of Exact and Natural Sciences, Jagiellonian University, Łojasiewicza 11, 30-348 Krakow, Poland; 3 II Department of Internal Medicine, Faculty of Medicine, Jagiellonian University Medical College, Skawińska 8, 31-066 Krakow, Poland; 4 Institute of Bioorganic Chemistry, Polish Academy of Sciences, Noskowskiego 12/14, 61-704 Poznan, Poland; pworsztynowicz@ibch.poznan.pl (P.W.); kpokrywka@ibch.poznan.pl (K.P.); sliwiak@ibch.poznan.pl (J.S.); mariuszj@amu.edu.pl (M.J.); 5 Center for the Development of Therapies for Civilization and Age-Related Diseases, Jagiellonian University Medical College, Skawińska 8, 31-066 Krakow, Poland; kamila.pietruszewska@uj.edu.pl (K.P.); z.mazurek@uj.edu.pl (Z.M.); anna.skalniak@uj.edu.pl (A.S.); 6 Department of Hematology and Bone Marrow Transplantation, Poznan University of Medical Sciences, Szamarzewskiego 84, 60-569 Poznan, Poland; lewandowski@ump.edu.pl; 7 Department of Crystallography, Faculty of Chemistry, Adam Mickiewicz University, Uniwersytetu Poznanskiego 8, 61-614 Poznan, Poland

**Keywords:** L-asparaginase, substrate affinity, leukemia, cell proliferation, cell apoptosis

## Abstract

L-asparaginases are used in the treatment of acute lymphoblastic leukemia. The aim of this work was to compare the antiproliferative potential and proapoptotic properties of novel L-asparaginases from different structural classes, viz. EcAIII and KpAIII (class 2), as well as ReAIV and ReAV (class 3). The EcAII (class 1) enzyme served as a reference. The proapoptotic and antiproliferative effects were tested using four human leukemia cell models: MOLT-4, RAJI, THP-1, and HL-60. The antiproliferative assay with the MOLT-4 cell line indicated the inhibitory properties of all tested L-asparaginases. The results from the THP-1 cell models showed a similar antiproliferative effect in the presence of EcAII, EcAIII, and KpAIII. In the case of HL-60 cells, the inhibition of proliferation was observed in the presence of EcAII and KpAIII, whereas the proliferation of RAJI cells was inhibited only by EcAII. The results of the proapoptotic assays showed individual effects of the enzymes toward specific cell lines, suggesting a selective (time-dependent and dose-dependent) action of the tested L-asparaginases. We have, thus, demonstrated that novel L-asparaginases, with a lower substrate affinity than EcAII, also exhibit significant antileukemic properties in vitro, which makes them interesting new drug candidates for the treatment of hematological malignancies. For all enzymes, the kinetic parameters (K_m_ and k_cat_) and thermal stability (T_m_) were determined. Structural and catalytic properties of L-asparaginases from different classes are also summarized.

## 1. Introduction

L-Asparaginases are enzymes that hydrolyze L-asparagine (L-Asn) to L-aspartic acid (L-Asp) and ammonia. The history of using L-asparaginases in leukemia therapy has its beginning in 1953, when Kidd reported the anticancer activity of guinea pig (*Cavia porcellus*) serum fractions containing L-asparaginase [1,2]. The guinea pig protein has never been approved for clinical use, instead, periplasmic L-asparaginase from *Escherichia coli* (EcAII) has been used clinically for the treatment of acute lymphoblastic leukemia (ALL) since 1978 [3]. The EcAII acts by depleting the circulating pool of L-Asn, an amino acid essential for the survival of some leukemic cells that lack the ability to synthesize L-Asn. Its targeted action spares the normal cells, which are able to synthesize L-Asn [4]. Recent reports indicate that L-asparaginases can be used not only in leukemia therapy but also in the treatment of other types of cancers [4,5,6,7].

**Figure 1:**
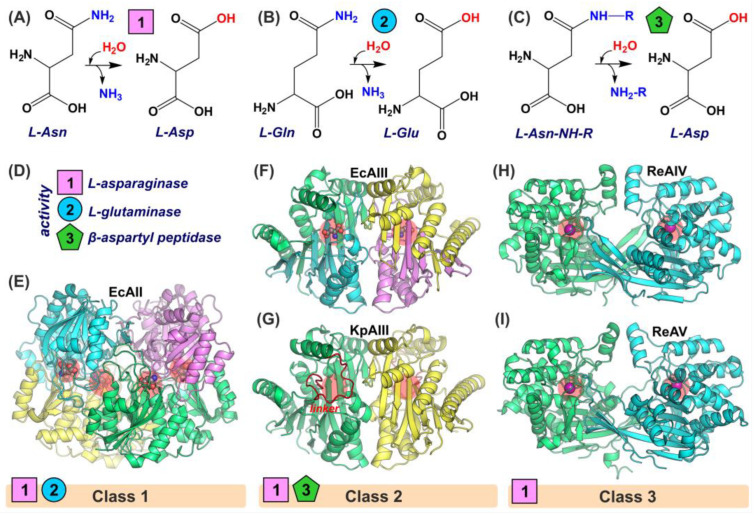
Catalytic properties and structures of L-asparaginases. Reaction catalyzed by enzymes with (**A**) L-asparaginase activity, (**B**) L-glutaminase (co-)activity, and (**C**) β-aspartyl aminopeptidase (co-)activity (NH_2_-R—amino acid residue). (**D**) Symbols of different activities presented in panels (**A**–**C**). (**E**) Structure of EcAII from *E. coli* (PDB: 6v23). (**F**) Structure of EcAIII (PDB: 2zal) from *E. coli.* (**G**) AlphaFold2 model of KpAIII (*K. pneumoniae*) precursor (linker marked in red). (**H**) Structure of thermostable ReAIV (PDB: 8osw) and (**I**) thermolabile enzyme ReAV (PDB: 7os5) from *R. etli*. In all panels, protein subunits are shown in different colors. Red circles mark active sites with L-Asp in space-filling representation (panels (**E**,**F**)). Violet spheres (panels (**H**,**I**)) mark Zn^2+^ coordinated in the active sites of ReAIV and ReAV.

From the structural point of view, L-asparaginases can be divided into three structural classes [8,9] (Figure 1). Class 1 includes tetrameric enzymes [10] initially identified in bacteria, although their homologs are present in yeast [11] and mammals [12,13]. Class 1 contains constitutive proteins (cytoplasmic, type I) with low (mM) substrate affinity and periplasmic (secreted type II) enzymes with higher (μM) affinity for L-Asn [14]. Class 2 contains type III enzymes which can be divided into K-dependent and K-independent proteins [15]. Type III L-asparaginases belong to the Ntn-hydrolase family [16] and are produced as inactive precursors that develop catalytic activity during auto-maturation [17]. Mature class 2 L-asparaginases are (αβ)_2_ heterodimers (Figure 1) with (mM) substrate (L-Asn) affinity. Class 2 enzymes were discovered in all domains of life [18]. Class 3 contains constitutive (thermostable, type IV) and inducible (thermolabile, type V) enzymes originally identified in the *Rhizobium etli*. Type IV and type V enzymes are homodimers (Figure 1) with (mM) substrate affinity [19].

On the one hand, currently only type II (class 1) enzymes from *E. coli* (EcAII) and *E. chrysanthemi* (current taxonomic classification: *Dickeya dadantii*) (ErAII) are approved as drugs in ALL therapy [20]. On the other hand, the potential therapeutic use of other L-asparaginases (from class 2 or class 3) is often neglected as those enzymes were rejected several years ago due to their too-low substrate affinity [21], while in fact their antileukemic properties have never been tested. The aim of this work was to screen the antileukemic potential of recombinant L-asparaginases from different sources and structural classes: EcAIII and KpAIII (class 2 enzymes from *E. coli* and *K. pneumoniae*, respectively; Figure S1), and ReAIV and ReAV (class 3 proteins from *R. etli*). EcAII (class 1 *E. coli* enzyme) served as a reference in our studies. In parallel with the expression of wild-type enzymes, we produced an inactive EcAIII mutant used as a control protein (Control-P) in proliferative and apoptotic assays. The tested bacterial enzymes differ not only in their affinity for L-Asn but also in their molecular size and enzymatic properties (Figure 1). Investigations were performed with four human leukemia cell lines: acute lymphoblastic leukemia (MOLT-4), Burkitt’s lymphoma (RAJI), acute monocytic leukemia (THP-1), and acute promyelocytic leukemia (HL-60). We have observed that tested enzymes (EcAIII, KpAIII, and ReAIV), possessing lower substrate affinity (mM) than EcAII (μM), also exhibit significant antileukemic properties in vitro, which makes them interesting drug candidates for the treatment of hematological malignancies.

## 2. Results

### 2.1. Biochemical Properties of Tested L-Asparaginases

All studied L-asparaginases were expressed in the *E. coli* BL21 Gold (DE3) strain. This host was appropriate for the efficient production of EcAIII and KpAIII (yields up to 60 mg per 1 L of bacterial culture). The same was observed for the Control-P protein. An expression of up to 50 mg/L was also possible for the ReAV, while the production of ReAIV was less efficient (up to 10 mg/L). The most problematic was the expression of EcAII in this system, as we were able to obtain up to 2 mg of the protein from 1L of bacterial culture. Therefore, for the EcAII expression, BL21 (DE3) strain Δ*ansA/*Δ*ansB* [22,23] was used, increasing the expression level to 10 mg/L.

A visual inspection of the protein samples after purification and dialysis to PBS (see Materials and Methods) showed that all proteins, with the exception of EcAII (which precipitated), survived in soluble form and were enzymatically active. To stabilize EcAII in the PBS buffer, we tested several non-toxic additives: sucrose, sorbitol, glycine, choline, and L-arginine. Among them, the most efficient was glycine, as the addition of 100 mM glycine prevented EcAII precipitation. The nanoDSF (differential scanning fluorimetry in nanoscale) experiment showed that the addition of 100 mM of glycine increases the thermal stability (T_m_) of EcAII (Table S1). We also noticed that the KpAIII protein could not be deep frozen at −80 °C in PBS, as it immediately precipitated after thawing. However, the addition of 100 mM of glycine acted as a cryoprotectant, allowing the safe storage of KpAIII. We determined the T_m_ of all the enzymes in PBS. The most thermostable was ReAIV (83.24 °C), while its counterpart ReAV had the lowest T_m_ of 48.41 °C. We also observed that additives such as glycine increase T_m_ by about ~3 °C (Table S1).

Using the Nessler method, we determined the kinetic parameters of the L-Asn hydrolysis of class 2 and class 3 enzymes in PBS (pH 7.4) at 37 °C, finding the highest L-Asn affinity for ReAIV (7.16 ± 2.03 mM), and the lowest for KpAIII (42.95 ± 7.77 mM) (Table 1). The kinetic parameters of the EcAII hydrolysis of L-Asn were determined using ITC, as the Nessler method is not sensitive enough for micromolar K_m_ determination [24]. The parameters determined in PBS at 37 °C summarized in Table 1 are different from the values reported earlier for the kinetic assays at optimal conditions (Table S2).

The EcAIII and KpAIII proteins also display additional β-aspartyl peptidase activity. β-Aspartyl peptidase activity was monitored using the GOT (glutamate-oxaloacetate transaminase) method and *N*-β-L-aspartyl-L-phenylalanine methyl ester (β-L-Asn-PheMe) as a substrate. Determined kinetic parameters indicated that KpAIII has a higher affinity for the tested β-peptide than EcAIII (Table 2).

We also monitored the change in the specific activity of tested L-asparaginases after incubation in PBS at 37 °C for 24 and 48 h. Our data revealed that at the beginning of the experiment, the highest specific activity was observed for EcAII; however, it dropped by ~61% after 24 h of incubation (Figure S2). We also observed that the specific activity of ReAIV gradually decreased in time. Interestingly, the specific activity of class 2 L-asparaginases, EcAIII and KpAIII, increased gradually after each day of incubation (Figure S2).

### 2.2. Preparation of Enzymes for Cell Line Studies

Each purification batch of L-asparaginases (concentrated to 10–15 mg/mL) was tested for endotoxin level (LPS, lipopolysaccharide) using the LAL (limulus amebocyte lysate) method. The LPS content per mg of purified protein was in the range of 0.20–0.23 EU/Ml (0.02–0.05 EU/mg) for EcAII; 0.44–0.59 EU/mL (0.02–0.40 EU/mg) for EcAIII; and 0.18–0.40 EU/mL (0.01–0.04 EU/mg) for KpAIII. In the ReAV samples, the LPS level was in the range of 0.79–0.98 EU/mL (0.05–0.10 EU/mg), while for ReAIV it was 0.13–0.16 EU/mL (0.06–0.07 EU/mg).

Prior to the experiments with human cell lines, we also tested the stability of each enzyme in the cell culture media and its components. The EcAII, EcAIII, and KpAIII enzymes tolerated 48 h of incubation in culture media perfectly well. The behavior of the class 3 enzymes was more problematic. For ReAIV, we observed a slight precipitation in all tested conditions (24–48 h) at the highest concentration of the enzyme (1.0 mg/mL). This effect was not observed at lower concentrations (0.001, 0.01, and 0.1 mg/mL). The ReAV protein precipitated heavily at all concentrations used (0.01, 0.1, and 1.0 mg/mL) and in all tested conditions (Figure S3A). We used SDS-PAGE to assay the content of the precipitate. The analysis of the gels revealed that the precipitate contained only ReAV (when incubated in RPMI-1640 or RPMI-1640 with antibiotics and L-glutamine) or simultaneously ReAV and BSA when the incubation was performed in the presence of 10% FBS (Figure S3B). To test the ReAV stability in the cell culture, we performed its incubation with RAJI cells for 24 and 48h. Unfortunately, also in this case, ReAV precipitation was observed (Figure S3C,D). This unusual behavior at 37 °C disqualified ReAV from further experiments with leukemia cell lines.

### 2.3. Antiproliferative Effect of L-Asparaginases

**Figure 2:**
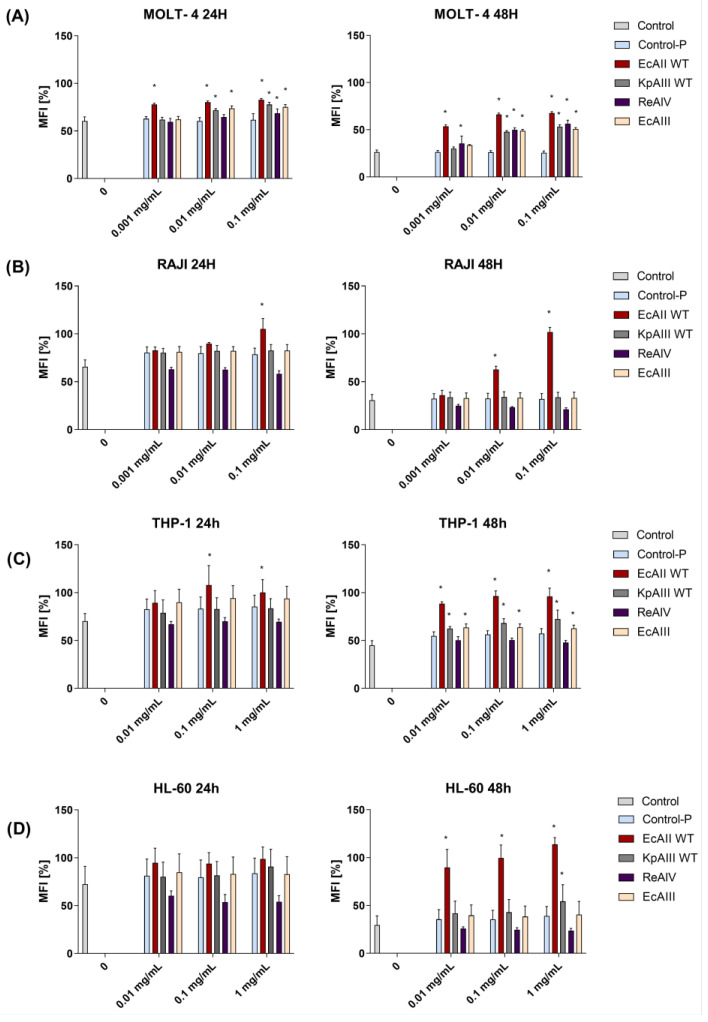
The effect of the tested enzymes on leukemic cell proliferation. Cells from the (**A**) MOLT-4, (**B**) RAJI, (**C**) THP-1, and (**D**) HL-60 lines were stained with PKH67 dye according to the manufacturer’s instructions. The maximum fluorescence was measured on day 0, and cells were then stimulated with the tested L-asparaginases for 24 and 48 h and analyzed using a flow cytometer (unstimulated cells and cells stimulated with the Control-P protein were used as controls). The presented results are from three independent experiments and are expressed as percentages of the mean (colored stripes) ± standard deviation (thin bar) of the maximum fluorescence value at day 0. * Marks a *p* < 0.05 probability according to Tukey’s post hoc test.

In the present study, we analyzed the antiproliferative effect of selected L-asparaginases on four common leukemia cell lines, HL-60, THP-1, RAJI, and MOLT-4. The experiments with MOLT-4 cells showed that all tested L-asparaginases (EcAII, EcAIII, KpAIII, and ReAIV) can inhibit cell proliferation after 48 h stimulation, but after 24 h, this effect was only detected for EcAII (all concentrations tested), KpAIII (0.01 mg/mL and 0.1 mg/mL), and ReAIV (only 0.1 mg/mL) (Figure 2A).

For the RAJI cells, the inhibition of proliferation was only observed for EcAII (0.1 mg/mL at 24 h incubation; 0.01 mg/mL and 0.1 mg/mL at 48 h) (Figure 2B). Analyses of the proliferation of THP-1 cells treated with the tested proteins showed that EcAII can inhibit cell proliferation after 24 h of incubation (0.1 mg/mL and 1 mg/mL; Figure 2C, left panel), and after 48 h, a similar effect was observed for all tested proteins except ReAIV (Figure 2C, right panel). In the case of the HL-60 cells, no effect on cell proliferation was observed after 24 h of incubation (Figure 2D, left panel), whereas a significant effect was observed after 48 h of incubation with the EcAII protein (all concentrations tested).

### 2.4. Proapoptotic Effect of L-Asparaginases

**Figure 3:**
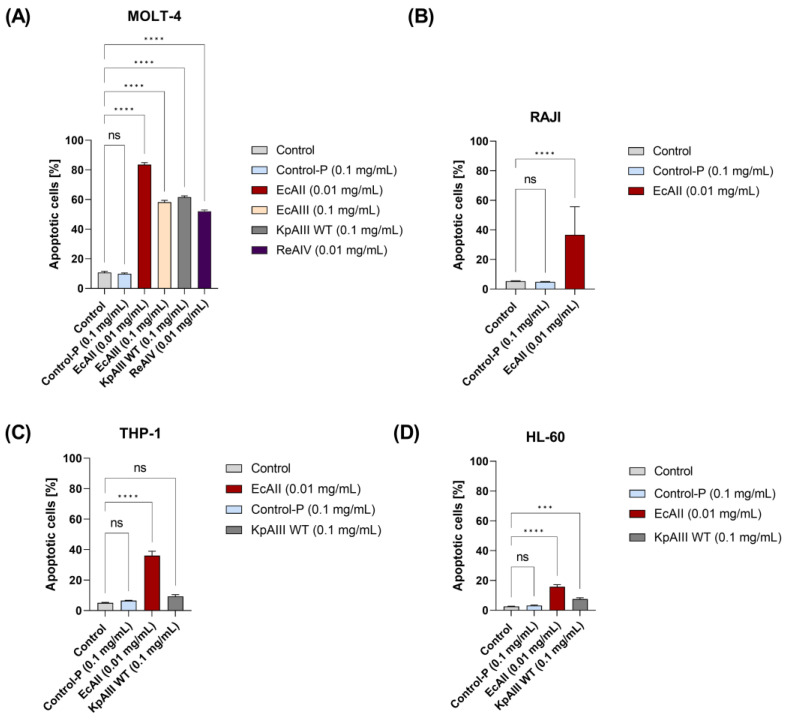
Influence of different L-asparaginases on the apoptosis of leukemia cell lines: (**A**) MOLT-4, (**B**) RAJI, (**C**) THP-1, and (**D**) HL-60 cells were cultured in the presence of selected enzymes in a full growth medium for 48 h, stained with a PE Annexin V Apoptosis Detection Kit I, and analyzed via flow cytometry. The L-asparaginases and their concentrations used in this experiment were based on the results from proliferation evaluation. The presented results are from three independent experiments and are expressed in Figure 2, i.e., as a percentage of the mean ± standard deviation of the dead cells (apoptotic and/or necrotic), *** marks a *p* < 0.005, and **** marks a *p* < 0.0001 probability according to Tukey’s post hoc test; ns: not significant. Gating strategies used in flow cytometry are in Figure S4.

Based on the above proliferation experiments, we selected proteins (and their concentrations) to assess their effect on the apoptotic process of leukemic cells. As shown in Figure 3A, in the case of the MOLT-4 cell line, all tested L-asparaginases showed a proapoptotic effect (Figure 3).

Percentage of apoptotic cells: EcAII (0.01 mg/mL) 86 ± 4%; EcAIII (0.1 mg/mL) 58.2 ± 4.1%; KpAIII (0.1 mg/mL) 61.5 ± 2.8%; ReAIV (0.01 mg/mL) 52 ± 3% vs. stimulated/negative control cells (Control-P protein 0.1 mg/mL) of 10.7 ± 2.6/9.9 ± 1.7% (Figure 3A). For the RAJI and THP-1 cells, the induction of apoptosis was confirmed only for the positive control protein EcAII (0.01 mg/mL); for the RAJI cells, the percentage of apoptotic cells was 36.7 ± 19% vs. 5.4 ± 0.7% for control cells and 4.9 ± 0.66% for the negative Control-P protein. For the THP-1 cells, the percentage of apoptotic cells was 36 ± 9% vs. 5.0 ± 0.9% for control cells and 6.5 ± 0.9% for the negative Control-P (Figure 3B,C). In the case of the HL-60 cells, a weak but significant effect was also observed for KpAIII at 0.1 mg/mL (7.5 ± 2.9% of apoptotic cells) and EcAII at 0.01 mg/mL (15.8 ± 4.5%), vs. control cells (2.4 ± 0.5%) and the negative Control-P protein (3.2 ± 0.9%) (Figure 3D).

## 3. Discussion

In this report, we have analyzed the antileukemic properties of novel L-asparaginases: EcAIII, KpAIII, and ReAIV. According to the literature data, the most important factor responsible for the therapeutic efficacy of L-asparaginases in ALL treatment is the substrate (L-Asn) affinity, which should be in the μM range. This range corresponds to the physiological level of L-Asn circulating in human blood, which varies between 40 and 80 μM [25]. One of the first reports indicating the K_m_ value that determines the in vivo activity of L-asparaginases was the analysis of the anticancer properties of *Pseudomonas geniculate* L-asparaginases [26]. Differences in the antitumor activity of two enzymes, A and AG, were related to their K_m_ values for L-Asn: 1 mM for enzyme-A and 15 μM for enzyme-AG. Only enzyme-AG (with L-glutaminase co-activity) showed antitumor activity in mice. Another factor favoring the antineoplastic activity of enzyme-AG was the relatively slow clearance from the plasma of tumor-bearing animals [26].

The L-asparaginases used in our studies differ not only in their 3D structure but also in their L-Asn affinity. In our experiments, we observed proliferation inhibition or cell death induction even in the presence of L-asparaginases, such as KpAIII, with K_m_ values significantly lower than for EcAII, which is currently used as a therapeutic enzyme [22,27,28]. Although PBS is not the optimal environment for activity studies for most of our enzymes (Table S2), our data indicated that all proteins are still active enough at physiological conditions to affect the survival of the leukemia cells. Our results indicate unambiguously that substrate (L-Asn) affinity alone, however important, should not be a discriminatory factor for testing new L-asparaginases being potential drug candidates. Another factor that should be considered is the stability of the enzyme, as proteins like ReAV with low T_m_ are not suitable for in vitro and further in vivo tests. Interestingly, the lack of antileukemic activity of the cytosolic *E. coli* L-asparaginase EcAI (class 1, type I) was not attributed to its low L-Asn affinity (K_m_ 3.5 mM) [29] but rather to the rapid clearance of the protein from the serum (very short half-life) or to the inactivation of the enzyme under physiological conditions since no activity was detectable in the serum after injection [30]. Therefore, enzymes should also retain their specific activity long enough to efficiently deplete the serum pool of L-Asn. Among the proteins used in this study, the most active enzyme, EcAII, lost its activity very quickly (24h). The same was observed for ReAIV; however, this protein needs exogenous Zn^2+^ to maintain its activity [31]. Interestingly, the specific activity of EcAIII and KpAIII increased during 48h of incubation. We can link this phenomenon to the time-dependent degradation of the linker which remains after maturation and covers the access to the active site preventing substrate binding [16]. The L-asparaginase activity might be also affected by additives present in protein preparation or by the components of cell culture media. In our studies, we used glycine as an additive to EcAII and KpAIII, as among the tested chemicals (see Section 2.1), only glycine effectively prevented EcAII precipitation. However, the 100 mM of glycine was diluted 10 to 100 times in the cell culture assays, which contain numerous other ingredients, e.g., all amino acids, vitamins, sugars, salts, and proteins. Therefore, the presence of extra glycine additive has a rather minor inhibitory effect in comparison with the impact of all the chemical components in the cell culture medium. Our studies also revealed that glycine itself did not affect the survival of the leukemia cells. Recombinant enzymes should also have acceptable LPS levels. All proteins produced in our experiments had LPS content in the range similar to that reported for pharmaceutical preparations of L-asparaginases [32,33].

Acute lymphoblastic leukemia cells are sensitive to L-asparaginase treatment because of their poor ability to synthesize sufficient amounts of endogenous L-Asn due to low expression levels of L-asparagine synthetase (ASNS) [34]. The results of our experiments with the MOLT-4 cell line are in line with previous observations confirming the efficacy of EcAII (e.g., Spectrila) in inhibiting proliferation or inducing apoptosis in vitro and in vivo [22,35,36]. Moreover, our data showed that other L-asparaginases such as EcAIII, KpAIII, and ReAIV are very promising agents in the field of anti-ALL therapy, as they induce apoptosis in MOLT-4 cells with only slightly lower rates than EcAII, and their proapoptotic potential is proportional to their K_m_ values. Although EcAIII, KpAIII, and ReAIV have lower substrate affinity than EcAII, their proapoptotic and antiproliferative action also seems to be related to the depletion of L-Asn in cell culture media, although this biological activity might require longer time or higher doses of the enzymes than in the case of EcAII. Additional L-glutaminase or β-aspartyl peptidase co-activities might also contribute to the biological effects, although these data need further verification, especially in the cases of EcAIII or KpAIII. Among the studied enzymes, ReAIV seems to be the most promising candidate for potential therapeutic use. The tested Ntn-hydrolases, EcAIII and KpAIII, needed a ~10-fold higher concentration than ReAIV to induce apoptosis at the same period of time. We also observed that some proteins, e.g., EcAIII, require more time (48h) and a higher concentration (0.1 mg/mL) to inhibit the proliferation of MOLT-4 cells in a mode similar to EcAII.

In experiments with Burkitt’s lymphoma cell line RAJI, some effect on apoptosis and proliferation was observed only for L-asparaginase with the lowest K_m_ for L-Asn, i.e., EcAII. Burkitt’s lymphoma is a non-Hodgkin’s lymphoma. It is a relatively rare cancer but is highly aggressive, resulting in rapidly growing tumors [37]. There are only a few reports discussing the use of L-asparaginases in the treatment of Burkitt’s lymphoma [38,39,40,41,42], presenting contradictory results. According to the most recent studies, it appears that the use of L-asparaginase as a single agent does not have the desired effect in this cell line. Although our experiments do not allow us to unequivocally confirm such a hypothesis, similar observations have already been demonstrated in in vivo studies in mice (engrafted with RAJI cells), which revealed that EcAII as a single agent showed little antitumor activity, but in combination with etoposide significantly suppressed tumor burden and improved survival time compared to the control group [43].

The sensitivity of other types of leukemia cells to L-asparaginases is usually lower than that of ALL cells. Therefore, we decided to investigate the effect of EcAII, EcAIII, KpAIII, and ReAIV on the proliferation of THP-1 and HL-60 cell lines. Of the enzymes tested, EcAII showed the best results in terms of the inhibition of the proliferation and induction of apoptosis in THP-1 and HL-60 cell lines. The observed weak proapoptotic activity of EcAII in HL-60 cells is in agreement with the previous finding that promyelocytes are resistant to L-asparaginase (EcAII) [44]. On the other hand, it was shown that acute myeloid leukemia (AML) THP-1 cells require L-Gln to survive [45]. This is also in agreement with our observations, as among the tested enzymes, i.e., EcAII, EcAIII, KpAIII, and ReAIV, only EcAII exhibits L-glutaminase co-activity with K_m_ for L-Gln in the range of 3.50–3.95 mM [22,27]. The EcAII counterpart from *E. chrysanthemi* (ErAII) has a lower affinity for L-Asn (K_m_ 47–58 μM) and slightly higher for L-Gln (K_m_ 0.36–6.70 mM) [46,47]. It was demonstrated that ErAII, which has higher L-glutaminase co-activity than EcAII, was more effective in the treatment of patients with AML [48]. L-Gln is essential for protein biosynthesis and cell cycle progression, and increased levels of L-Gln may enhance L-Asn biosynthesis via ASNS [49]. On the other hand, the depletion of L-Gln simultaneously with L-Asn, during ALL therapy with the use of L-asparaginase EcAII (carrying L-glutaminase co-activity), increases side effects related to hepatotoxicity, pancreatitis, neurotoxicity, hyperammonemia, hyperglycemia, leukopenia, thrombosis, and bleeding [5,50]. Novel L-asparaginases tested in this study (EcAIII, KpAIII, and ReAIV) are free of L-glutaminase co-activity; therefore, their potential application in leukemia therapy will prevent the occurrence of side effects related to L-Gln depletion. However, EcAIII and KpAIII carry additional β-aspartyl peptidase co-activity, but at this moment there are no data concerning how such a co-activity may influence leukemia cells.

According to our knowledge, there are only a few reports on antiproliferative or proapoptotic effects induced by novel source L-asparaginases in AML (e.g., THP-1) and promyelocytic (e.g., HL-60) cell lines. It has been reported, for example, that L-asparaginase from *Burkholderia pseudomallei* (GenBank: ABA50799.1, class 1, type II), which has no detectable L-glutaminase activity, affects the viability of THP-1 cells [51]. As THP-1 cells are more sensitive to the depletion of L-Gln than L-Asn, these data conflict with our observations. Unfortunately, the K_m_ values for L-Asn and L-Gln were not determined for the *B. pseudomallei* enzyme, making it difficult to assess the origin of the cytotoxic activity against the THP-1 cells. In another study, it was shown that *Mycobacterium tuberculosis* L-asparaginase (class 1, type I, Uniprot: P9WPX5) with a K_m_ of 8.36 mM did not induce any morphological changes or cellular toxicity when incubated with THP-1 cells [52], and these data are similar to our observations for the enzymes with millimolar K_m_ and no L-glutaminase co-activity. On the other hand, the L-asparaginase from *Pyrococcus furiosus* (K_m_ 1.623 mM, no L-glutaminase activity, Uniprot: Q8U4E6), belonging to class 2 type III (the same class/type as for EcAIII or KpAIII), was reported to induce apoptosis of THP-1 cells [53]. It was shown that an enzyme isolated from the culture supernatant of *Enterobacter cloacae* (K_m_ 1.58 mM) exhibited antiproliferative activity in HL-60 and MOLT-4 cells [54]. The cytotoxicity (IC_50_) against HL-60 was comparable to that of the commercial EcAII L-asparaginase, while the same effect against MOLT-4 was lower than that observed for EcAII. The sequence of the *E. cloacae* enzyme was not provided in this paper, but SDS-PAGE analysis showed the molecular weight (M_w_) of a monomer to be 52 kDa. These data are rather confusing, as sequence databases list three L-asparaginases from *E. cloacae* (encoded by the genes ansA, ansB, and iaaA), but these proteins have M_w_ values in the range of 32–36 kDa. An unusual M_w_ of 63–65 kDa has also been reported for L-asparaginase from *Bacillus licheniformis* (K_m_ 1.518 µM), which showed remarkable antiproliferative activity against HL-60 cells [55]. However, this information is not supported by sequence databases, as the *B. licheniformis* genome does not encode an L-asparaginase with such a high M_w_. It was also shown that an enzyme isolated from soybean root nodules (K_m_ for L-Asn 0.36 mM) had higher antiproliferative activity in MOLT-4 and HL-60 cells than EcAII [56]. It is possible that the extracted protein was a thermolabile class 3 L-asparaginase. In a report suggesting that *Yarrowia lipolytica* L-asparaginase inhibited the proliferation and growth of MOLT-4 and RAJI cells [57], the authors claimed that they investigated type II (secreted) L-asparaginase (K_m_ not determined), but the sequence [58] indicated that the tested enzyme was an Ntn-hydrolase. In a report about anticancer properties of the Pyrococcus abyssi L-asparaginase [59], the authors described the enzyme as “L-asparaginase I”. However, the provided K_m_ value of 2.05 mM, sequence, and structure model unambiguously indicate a class 2 Ntn-hydrolase. Therefore, it appears that aside from our studies on the EcAIII, KpAIII, and ReAIV enzymes, there are also other reports discussing potential antileukemic properties of class 2 and class 3 L-asparaginases; however, those L-asparaginases were incorrectly described or classified.

## 4. Materials and Methods

### 4.1. Enzyme Expression and Purification

Genes encoding the following L-asparaginases: EcAII (Uniprot: P00805), EcAIII (Uniprot: P37595), and KpAIII (Uniprot: A6T6S6), were synthesized using GenScript and cloned to vectors carrying N-terminal 6xHis-tag (pET28a or pMSCG92). The synthetic gene of ReAIV (GeneArt; Uniprot: Q2KB35) and the gene-encoding ReAV (Uniprot: Q2K0Z2; obtained from German Collection of Microorganisms and Cell Cultures GmbH) were cloned to the pET151D-TOPO vector as described previously [19,60]. All proteins were expressed in *E. coli* BL21 Gold (DE3), except EcAII, which was produced in another *E. coli* strain, BL21 (DE3) Δ*ansA/*Δ*ansB*, designed for the expression of highly active L-asparaginases [22,23]. We also produced an inactive EcAIII mutant carrying the V208A/G209Q/P212S substitutions. This triple mutant was generated via random mutagenesis [61] and was used as a control protein (Control-P) in proliferative and apoptotic assays.

The expression of EcAIII and KpAIII (and Control-P) was carried out at 37 °C in LB medium. When OD_600_ reached ~1.00, the cultures were cooled down to 18 °C, and protein expression was induced with 0.5 µM IPTG. The cultures were grown overnight at 18 °C. The expression of ReAIV and ReAV was carried out as described previously [19,60]. After overnight expression, bacterial cells were centrifuged and re-suspended in 50 mM of Tris-HCl buffer at a pH of 8.0, containing 500 mM of NaCl, 20 mM (or 50 mM) of imidazole and (optionally) 10% glycerol. Bacteria were disrupted using sonication, and centrifuge-clarified lysates were loaded on a Ni-NTA Agarose column (Macherey-Nagel, Düren, Germany). Unbonded proteins were washed two times with buffer A (50 mM of Tris-HCl at a pH of 8.0, 500 mM of NaCl, and 20 mM or 50 mM of imidazole). The 6xHis-tagged proteins were eluted from the column with buffer B (50 mM of Tris-HCl at a pH of 8.0, 500 mM of NaCl, and 400 mM (or 500 mM) of imidazole, 10% glycerol). Protein purity and homogeneity were checked with SDS-PAGE (Figure S5).

### 4.2. Preparation of Proteins for Human Cell Line Studies

The following enzymes: EcAII, EcAIII, ReAIV, ReAV, and Control-P, were concentrated to 10–15 mg/mL at 4 °C using Amicon centrifugal filters (MWCO 10 kDa, Merck KGaA, DE). KpAIII and ReAIV were concentrated using a dialysis tube and PEG20000 as described elsewhere [62]. After concentration, enzymes (in 1–3 mL volume) were transferred to mini dialysis devices (Slide-A-Lyzer, MWCO 10 kDa, Thermo Fisher Scientific, USA) and dialyzed overnight at 4 °C to sterile PBS (phosphate-buffered saline) buffer (Adlab, Poland) with three exchanges of the buffer volume. After dialysis, proteins were collected, filtered via a sterile 0.22 μm syringe filter, placed in sterile Eppendorf tubes, and immediately frozen at −80 °C.

For EcAII, which precipitated during overnight dialysis to PBS, different stabilizing additives, such as sucrose, sorbitol, glycine, choline, and L-arginine [63,64,65], were tested at a 100 mM concentration. The additive stocks of 1000 mM were prepared in sterile PBS and filtered with a 0.22 μm syringe filter. The same additives were tested as freezing stabilizers for KpAIII, which could not be frozen for long-term storage at −80 °C in PBS, as it precipitates after thawing. All proteins obtained from different purification batches were tested for lipopolysaccharide (LPS) content [66,67]. The LPS level was determined using a Pierce Chromogenic Endotoxin Quantification Kit (Thermo Fisher Scientific, Waltham, MA, USA).

### 4.3. Determination of Enzyme Kinetic Parameters and Thermal Stability

The kinetic parameters of EcAIII, KpAIII, ReAIV, and ReAV at 37 °C in PBS (Figure S6) as well as the change in L-asparaginase-specific activity after 24 and 48 h of incubation at 37 °C were determined using the Nessler method as described previously [68] at a protein concentration of 0.5–1.5 µM. The kinetic parameters of L-Asn hydrolysis with EcAII were determined using the ITC single-injection method (ITC-SIM) and PEAQ-ITC calorimeter (MALVERN); a 5 µL aliquot of 50 mM L-Asn dissolved in PBS buffer was injected into the reaction cell (of 200 µL volume), containing 50 nM of EcAII enzyme in PBS with an injection time of 8 s and at stirring speed of 750 rpm and at 37 °C. Differential power (DP) was set to 10 μcal s^−1^.

For EcAIII and KpAIII, β-aspartyl peptidase activity was determined using a continuous enzyme-coupled assay and substrate (*N*-β-L-aspartyl-L-phenylalanine methyl ester) at the concentration range 0.69–18 mM and protein concentration of 5–7 µM. Reactions were performed in PBS, containing 600 µM α-ketoglutarate, 190 µM NADH, 5 U of GOT (glutamate-oxaloacetate transaminase), and 3.5 U of MD (malate dehydrogenase). The NADH to NAD^+^ conversion was measured as a decrease in absorbance at 340 nm (Figure S7). Kinetic calculations were performed using the Enzyme Kinetics App included in OriginPro software v. 9.7.0.188 (OriginLab, Northampton, MA, USA).

The thermal stability of the enzymes was monitored via nanoDSF using a Prometheus Panta (NanoTemper Technologies, Munich, Germany) instrument. Melting scans were recorded by monitoring fluorescence emission at 330/350 nm for samples subjected to a 25–95 °C temperature ramp at 1 °C/min. As the enzymes used in our studies have variable thermal stability, they were tested for stability in the cell culture medium and its components prior to application to the human cell line cultures. Tests were carried out in sterile 96-well plates using different concentrations (0.01, 0.1 or 1.0 mg/mL) of enzymes added to the four tested solutions (100 μL) containing: (1) RPMI-1640 (PAN Biotech, Aidenbach, Germany); (2) RPMI-1640 with 10% FBS (PAN Biotech, Aidenbach, Germany); (3) RPMI-1640 with antibiotics (Antibiotic Antimycotic Solution, Merck, Darmstadt, DE) and L-glutamine (Merck, Darmstadt, DE); and (4) RPMI-1640 with antibiotics, L-glutamine and 10% FBS. The plate was incubated at 37 °C and inspected visually every 24 h for the occurrence of any precipitation.

### 4.4. Cell Proliferation

The proliferation assay for the leukemic cells was performed using the PKH67 dye and flow cytometry. Briefly, on the day of the experiment, cells from the tested lines (MOLT-4, RAJI, THP-1, and HL-60; all from ATCC, Manassas, VA, USA) were stained with PKH67 dye according to the manufacturer’s instructions (Sigma-Aldrich, Saint Louis, MO, USA). Next, a small number of cells (~10,000) were used to measure the maximum fluorescence (on day 0) and the rest of the cells were seeded on 24-well plates at a density of 20,000/well (final volume 250 µL) and cultured for 24–48 h in complete growth medium RPMI-1640 supplemented with 10% FBS and L-glutamine and antibiotics (PAN Biotech, Aidenbach, Germany and Sigma-Aldrich, Saint Louis, MO, USA) in the presence of the tested proteins at different concentrations (0.001–0.1 mg/mL for MOLT-4 and RAJI and 0.01–1 mg/mL for HL-60 and THP-1 lines). The concentrations of enzymes used with a particular cell line were optimized in preliminary experiments. After 24 and 48 h, the mean fluorescence intensity of cells was analyzed with flow cytometry (FACSCanto II BD Bioscience, San Jose, CA, USA). The results are presented as the mean values ± standard deviation (SD) of the percentage of the mean fluorescence intensity of cells from day 0 and were obtained from at least three independent experiments.

### 4.5. Cell Apoptosis

Based on the proliferation experiments, we selected proteins (and their concentrations) to assess their effect on apoptosis induction in leukemic cells. As in the assessment of proliferation, the apoptotic effect of the tested proteins on the HL-60, THP-1, RAJI, and MOLT-4 cell lines was assessed using flow cytometry. On the day of the experiment, cells were seeded at a density of 20,000/well in a 24-well plate and cultured in a complete growth medium in the presence of the tested proteins (proteins and their concentrations were selected based on the results of the proliferation experiment). The culture was maintained for 48 h. After this time, cells were centrifuged (500 × g/5 min), stained according to the manufacturer’s protocol using the PE Annexin V Apoptosis Detection Kit I (BD Pharmingen, San Diego, CA, USA), and analyzed using a FACSCanto II flow cytometer (BD Bioscience, San Diego, CA, USA). Cells negative for annexin V and 7-AAD were considered viable. Cells expressing annexin V or both annexin V and 7-AAD were classified as apoptotic.

The results are presented as the means value ± standard deviations of the percentage of apoptotic/necrotic cells and were obtained from at least three independent experiments (with two replicates for each condition); for gating strategies, see Supplementary Figure S4.

### 4.6. Statistical Analyses

The statistical analysis was performed using GraphPad Prism 9.0 software (GraphPad Software Inc., San Diego, CA, USA). All comparisons were performed using a one-way analysis of variance (ANOVA) with Tukey’s post hoc test. Descriptive statistics were presented as the means ± SD. Correlations between analyzed factors were calculated using Spearman’s rank method. Type I statistical error *p* < 0.05 was considered significant.

## 5. Conclusions

In the present study, we analyzed the antiproliferative and proapoptotic effects of new L-asparaginases on four leukemia cell lines, HL-60, THP-1, RAJI, and MOLT-4. The results indicate that the enzymes EcAIII, KpAIII, and ReAIV have unquestionable, albeit different (time-dependent and dose-dependent), potential in the inhibition of the proliferation or induction of the apoptosis of leukemia cells. Although the outcomes of our investigations are promising, some of the shortcomings of this study should also be highlighted.

Firstly, the results were obtained using commercially available cell lines that are “artificial systems” and might carry some genetic abnormalities. The biological effects of EcAIII, KpAIII, and ReAIV should also be investigated using cells isolated from leukemia patients. The actions of any enzyme should also be tested in vivo to verify its stability, immunogenicity, and antineoplastic properties. Currently, reports from studies in animal models are available only for the commercial enzymes EcAII or ErAII [69,70,71], and there are no data for Nth-hydrolases (class 2) or Rhizobium etli-type (class 3) proteins.

Secondly, we were focused on evaluating the antiproliferative and proapoptotic properties of EcAIII, KpAIII, and ReAIV, related to the primary activity of these enzymes, i.e., the hydrolysis of L-Asn. However, our studies suggested that enzymes with a secondary activity, e.g., L-glutaminase co-activity, might act more efficiently in selected leukemia subtypes. Class 2 L-asparaginases, e.g., EcAIII, are free of L-glutaminase co-activity but demonstrate β-aspartyl aminopeptidase co-activity [72]. With no studies on the effect of the removal of the toxic β-aspartyl dipeptides on the survival of leukemia cells, it is difficult to predict how such co-activity will affect cancer (or healthy) cells. So far, there are no data about any secondary activity of class 3 L-asparaginase ReAIV. However, considering their unusual catalytic center with a Zn^2+^ [60], we cannot exclude that ReAIV could also catalyze other biochemical reactions.

As the long-term goals of our studies are devoted to developing new therapeutic enzymes, optimizing treatment protocols, and expanding clinical applications of L-asparaginases, there is no doubt that comprehensive knowledge about the mode of action through which enzymes from classes 2 and 3 modulate the leukemia cell behavior will be of high importance for designing innovative cancer therapies. It can also be concluded that the inconsistencies of the available experimental data about the possibility of the use of L-asparaginase in the treatment of non-ALL leukemias need to be verified and systematized to define clear recommendations.

## Figures and Tables

**Table 1:** Kinetic parameters of L-Asn hydrolysis at 37 °C in PBS (pH 7.4).

Enzyme	K_m_ [mM]	k_cat_ [s^−1^]	k_cat_/K_m_ [mM^−1^s^−1^]
EcAII	0.012 ± 0.002	39 ± 1	3301 ± 561
EcAIII	17.66 ± 1.87	5.77 ± 0.19	0.33 ± 0.10
KpAIII	42.95 ± 7.77	10.44 ± 0.96	0.24 ± 0.12
ReAIV	7.16 ± 2.03	9.98 ± 0.61	1.39 ± 0.48
ReAV	24.81 ± 3.22	28.53 ± 1.60	1.15 ± 0.21

**Table 2:** Kinetic parameters for β-L-Asn-PheMe hydrolysis at 37 °C in PBS (pH 7.4).

Enzyme	K_m_ [mM]	k_cat_ [s^−1^]	k_cat_/K_m_ [mM^−1^s^−1^]
EcAIII	3.57 ± 0.58	0.112 ± 0.007	0.031 ± 0.007
KpAIII	2.44 ± 0.47	0.017 ± 0.003	0.007 ± 0.003

## Data Availability

Data are contained within the article and Supplementary Materials.

## Supplementary Materials

The following supporting information can be downloaded at https://www.mdpi.com/article/10.3390/molecules29102272/s1: Table S1: Thermal stability of the tested enzymes in PBS (pH 7.4); Table S2: Kinetic parameters of tested enzymes at optimal conditions; Figure S1: Alignment of KpAIII and EcAIII sequences; Figure S2: Specific activity (U/mg) of the tested enzymes after 24 and 48 h incubation in PBS at 37 °C; Figure S3: Stability of enzymes in the cell culture media and its components; Figure S4: Gating strategy used in flow cytometry analysis of leukemia cell apoptosis; Figure S5: SDS-PAGE gels from protein purification; Figure S6: Fitting of kinetic data obtained using the Nessler method to the Michaelis–Menten equation; Figure S7: Fitting of kinetic data obtained using the GOT method to the Michaelis–Menten equation.

## Abbreviations and Symbols

ALL, acute lymphoblastic leukemia; AML, acute myeloid leukemia; ASNS, L-asparagine synthetase; BSA, bovine serum albumin; FBS, fetal bovine serum; EcAII, *E. coli* type II L-asparaginase (class 1, periplasmic); EcAIII, *E. coli* type III L-asparaginase (class 2, Ntn-hydrolase); KpAIII, K. pneumoniae type III L-asparaginase (class 2, Ntn-hydrolase); ReAIV, R. etli type IV L-asparaginase (class 3, thermostable); ReAV, R. etli type V (class 3, thermolabile).
