# Supplementary material for: Substrate Affinity Is Not Crucial for Therapeutic L-Asparaginases: Antileukemic Activity of Novel Bacterial Enzymes
Source: Molecules. 2024 May 11;29(10):2272. doi: 10.3390/molecules29102272 (PMC11124013; doi:10.3390/molecules29102272)
Supplement: Supplementary file 1 [file molecules-29-02272-s001.zip › molecules-2981070-supplementary.pdf]

SUPPLEMENTARY MATERIALS (figures and tables)

Table S1. Thermal stability of the tested enzymes in PBS (pH 7.4).

Enzyme	Additive 100 [mM]	T_m [°C]
EcAIII	-	74.81
KpAIII	-	75.00
	glycine	78.40
	choline	74.94
	sorbitol	75.12
	sucrose	75.13
	arginine	44.96
ReAIV	-	83.24
ReAV	-	48.41
EcAII	-	63.50
	glycine	67.60
	choline	63.70
	sorbitol	64.10
	sucrose	64.60
	arginine	60.50

Table S2. Kinetic parameters of tested enzymes in optimal conditions.

Enzyme	K_m [mM]	k_{cat} [s^{-1}]	k_{cat}/K_m [$mM^{-1}s^{-1}$]
EcAII*	optimal conditions: 23 mM Tris-HCl buffer pH 8.5		
	0.018 ± 0.002	60 ± 7	3333 ± 370
EcAIII	optimal conditions: 50 mM Tris-HCl buffer pH 8.5 200mM NaCl		
	22.4 ± 2.1	8.3 ± 0.3	0.38 ± 0.05
KpAIII	optimal conditions: PBS, 37°C		
	the best kinetic parameters were determined in PBS as presented in Table 1 in the main text		
ReAIV**	optimal conditions: 20 mM Tris-HCl buffer pH 9.0, 1.0 μM Zn^{2+}, 37°C		
	1.5 ± 0.1	770 ± 17	526 ± 34
ReAV**	optimal conditions: 20 mM Tris-HCl buffer pH 9.0, 2.5 μM Zn^{2+}, 37°C		
	2.1 ± 0.2	603 ± 24	294 ± 31

*Data from Anishkin et al. (2015) J Mol Biol. 427:2867-85
**Data from Sliwiak et al. (2024) Front in Chem 12:1373312

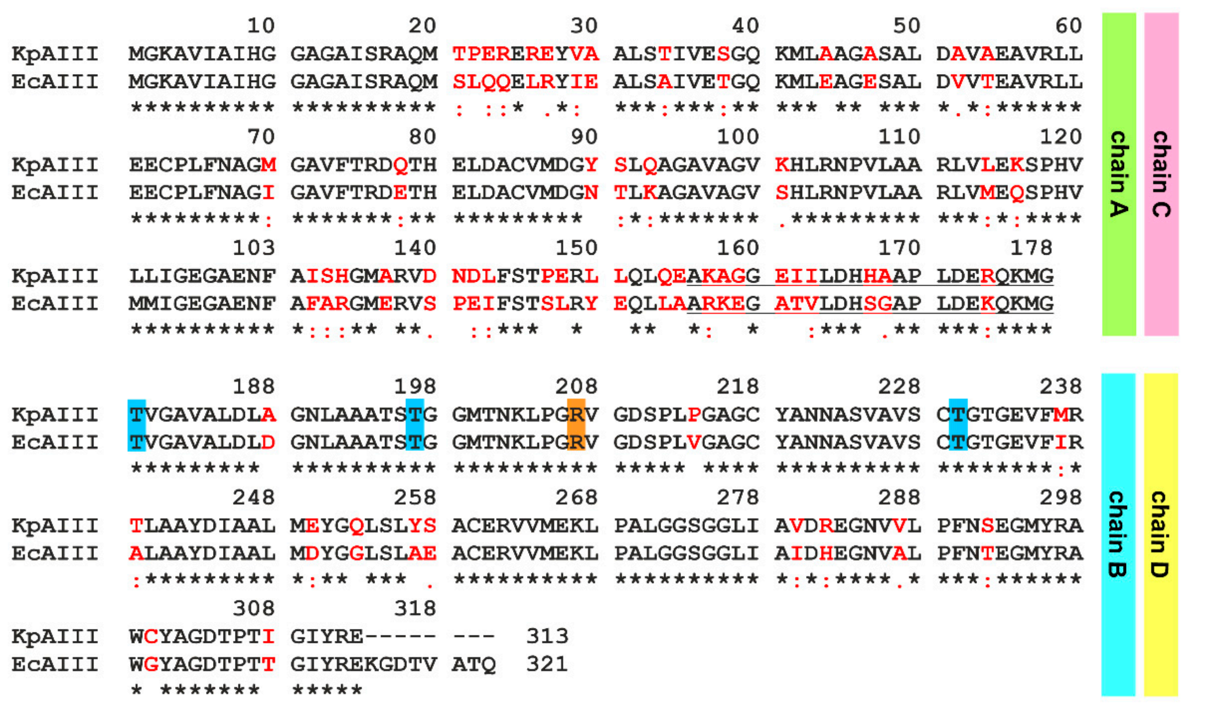

Figure S1. Alignment of KpAIII and EcAIII sequences. Red color marks different residues. Asterisks (*) marks identical (conserved) residues, colon (:) indicates different residues with similar chemical properties, blank space ( ) indicates residues with different chemical character, period (.) indicates a semi-conservative substitution. Residues that are part of the flexible linker are underlined. Blue boxes indicate a conserved threonine triplet (Thr179, Thr197, Thr230), orange box marks Arg207 which is crucial for substrate binding. Elongated color boxes mark protein chains (the same as in Fig. 1) that create the complete KpAIII and EcAIII enzymes.

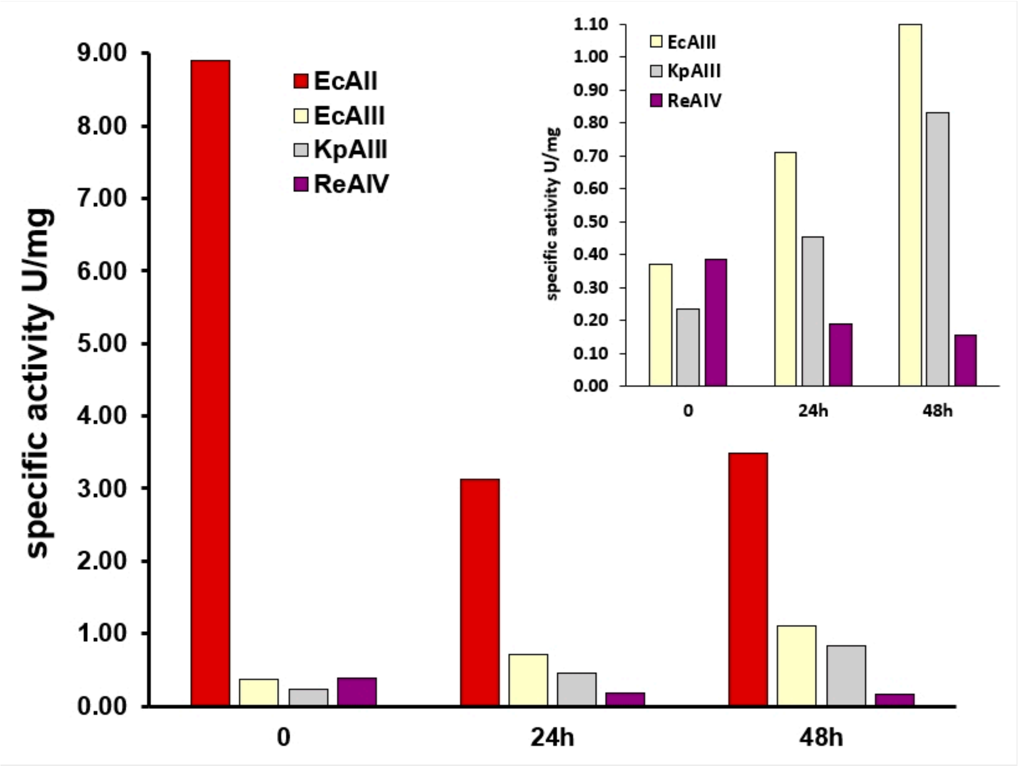

Figure S2. Specific activity (U/mg) of the tested enzymes after 24 and 48 h incubation in PBS at 37°C. The highest activity was observed for EcAII protein, while lower for EcAIII, KpAIII and ReAIV enzymes. Inset presents comparison of the data obtained only for EcAIII, KpAIII and ReAIV (without EcAII). Tests were performed using Nessler method and 24 mM substrate concentration.

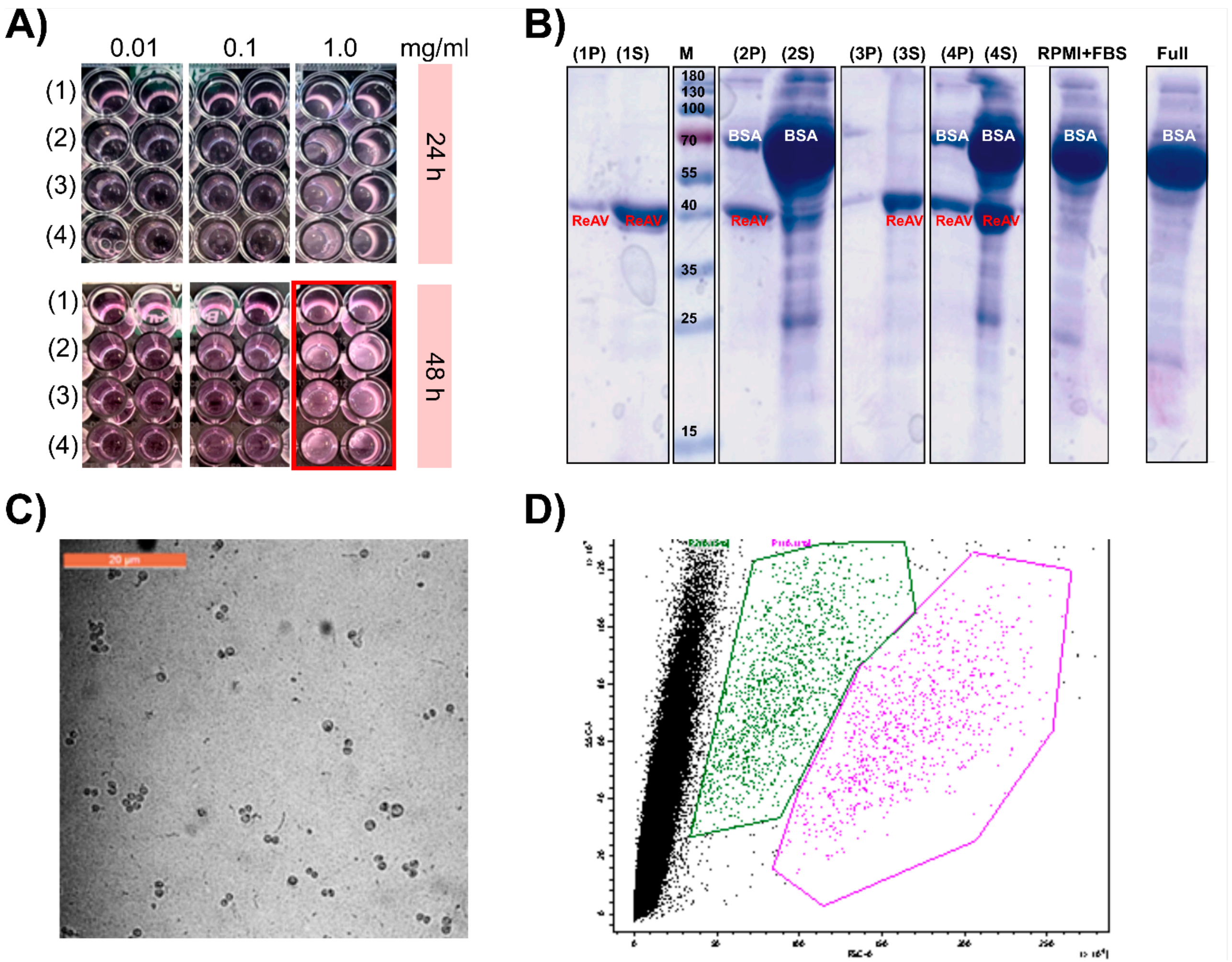

Figure S3. Stability of enzymes in the cell culture media and its components. (**A**) Visual inspection of precipitation of ReAV protein after 24 and 48 h of incubation in different solutions (each condition in double repeat): (1) RPMI-1640, (2) RPMI-1640 with 10% FBS, (3) RPMI-1640 with antibiotics and L-glutamine, and (4) full medium: RPMI-1640, antibiotics, L-glutamine and 10% FBS; in each condition white precipitate (in suspension) proportional to ReAV concentration was visible; samples collected after 48 h of incubation with the highest concentration of ReAV (1.0 mg/ml) were analyzed by SDS-PAGE (red frame). (**B**) The SDS-PAGE analysis of centrifuged precipitate (P) and supernatant (S) collected from solutions (1) – (4). (**C**) Sample micrograph of the culture of the RAJI cells after 24 h incubation with the ReAV in concentration 1 mg/ml: large spherical objects are RAJI cells, while scattered granular bodies visible in the background of the picture is the suspension of ReAV precipitate. (**D**) Representative dot plot of RAJI cells incubated for 24 h with the highest concentration of ReAV and analyzed by flow cytometry; purple - live cells, green - apoptotic/necrotic cells, black - ReAV precipitate.

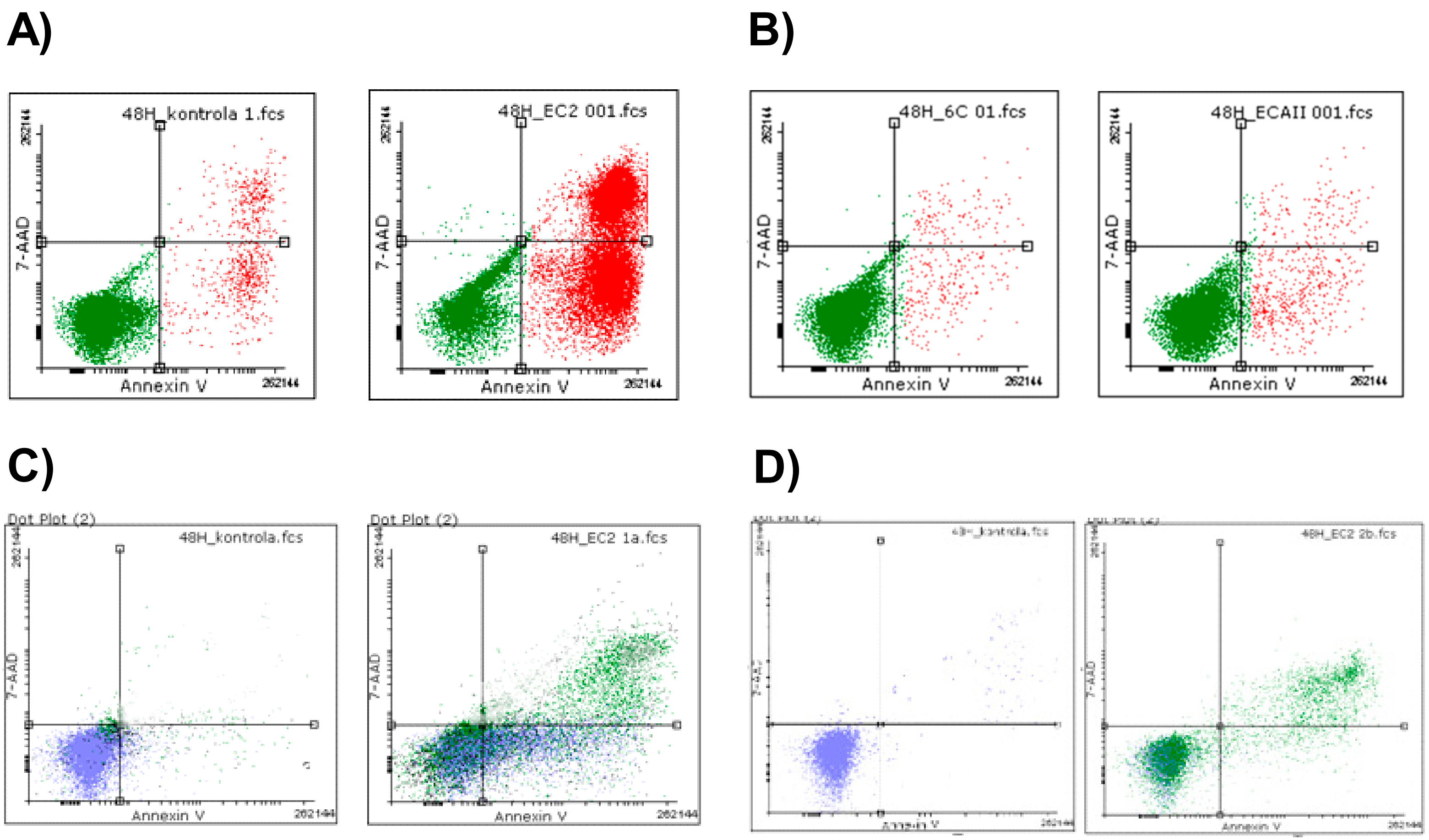

Figure S4. Gating strategy used in flow cytometry analysis of leukemia cell apoptosis. Cells were gated to determinate apoptosis by annexin V/7-AAD staining. Live cells are located in the lower left quadrant (annexin V -/7AAD -), while early apoptotic (annexin V +/7AAD -) and late apoptotic/necrotic (annexin V +/7AAD +) cells are located in the lower right and upper right quadrant, respectively. Representative dot plots (annexin V/7-AAD) of: (**A**) non-stimulated (left plot) and EcAII stimulated (48 h, right plot) MOLT-4, (**B**) RAJI, (**C**) THP-1 and (**D**) HL60 cells.

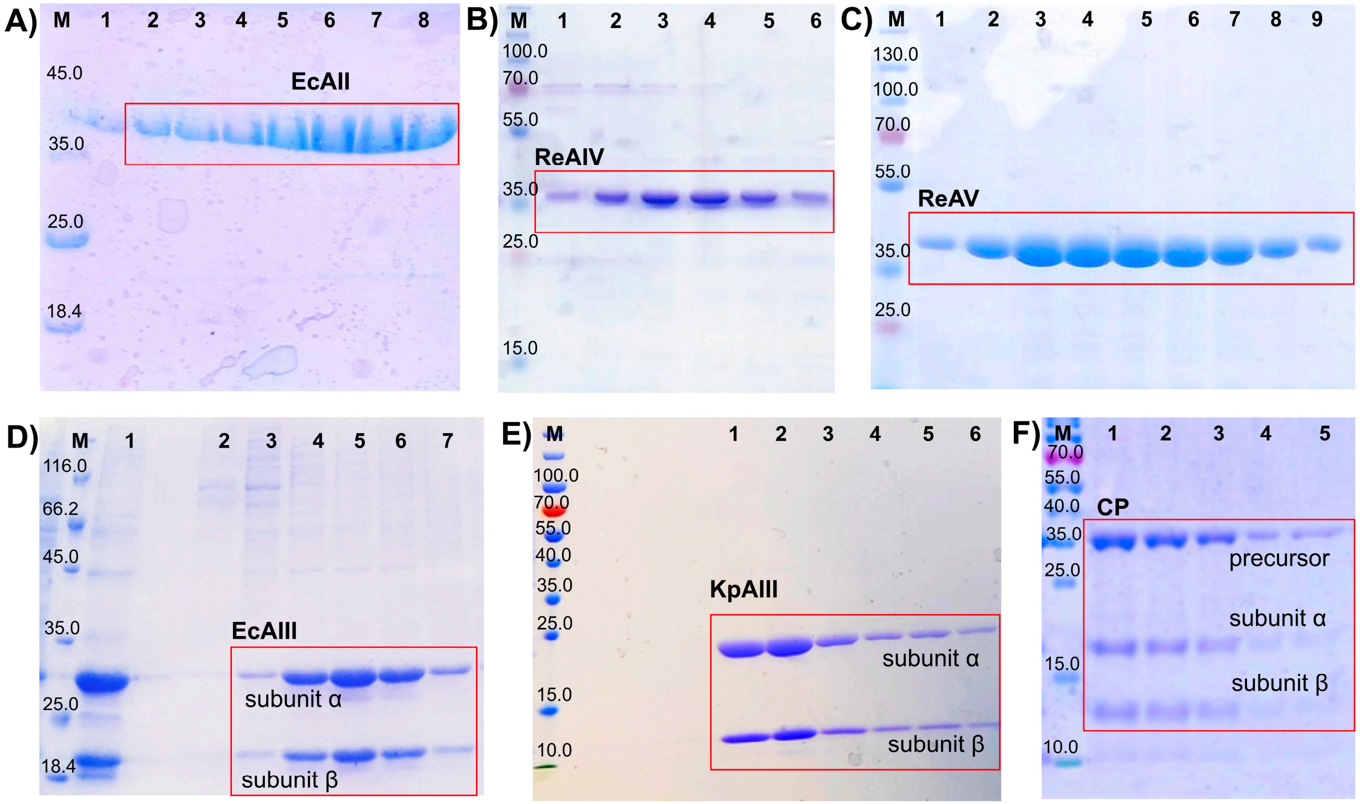

Figure S5. SDS-PAGE gels from protein purification. (**A**) EcAII after affinity chromatography (lines 1-8). (**B**) ReAIV after size exclusion chromatography (SEC; lines 1-6); (**C**) ReAV after SEC (lines 1-9). (**D**) EcAIII after affinity chromatography (lines 3-7). (**E**) KpAIII after affinity chromatography (lines 1-6). (**F**) Control protein (CP) after affinity chromatography (lines 1-6).

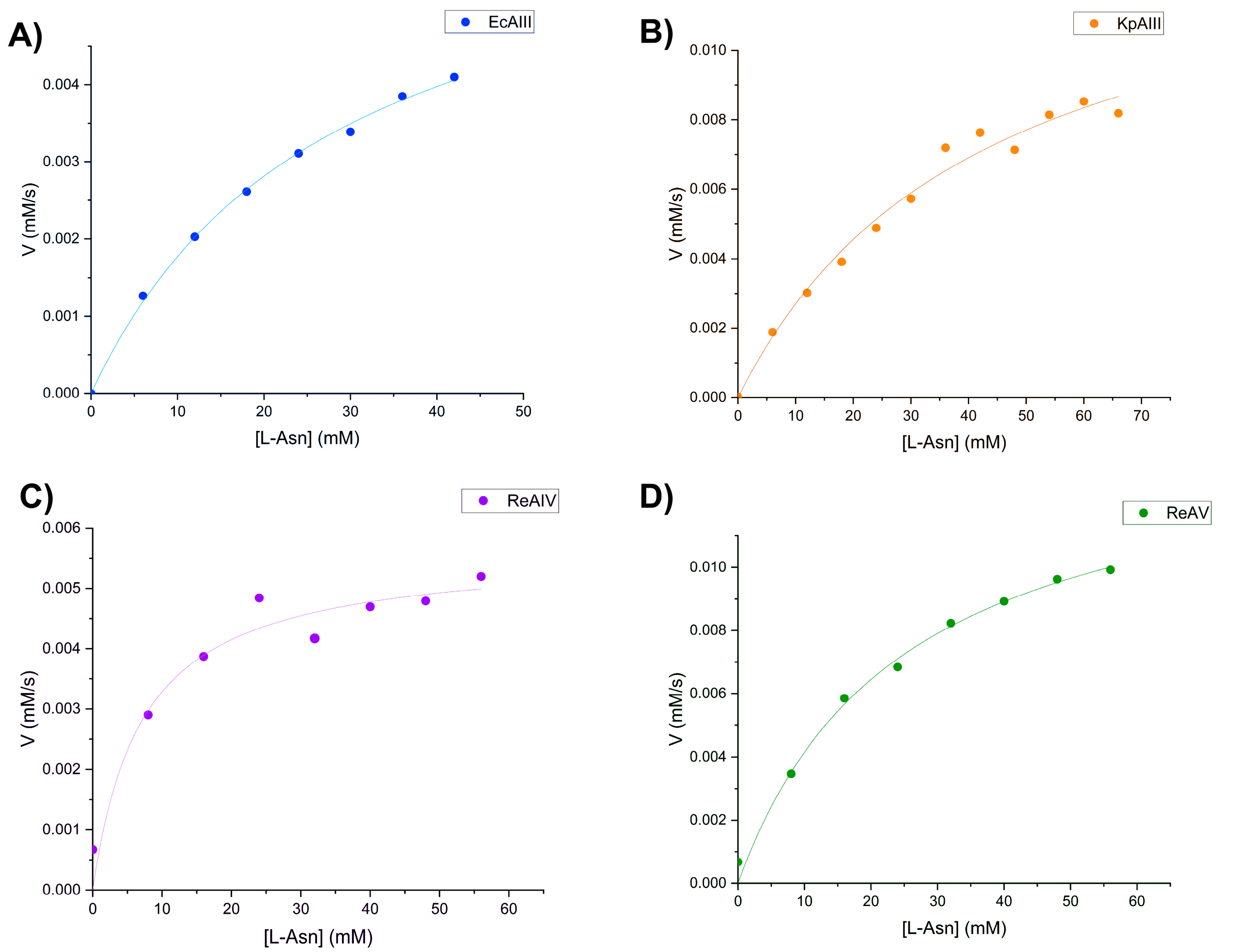

Figure S6. Fitting of kinetic data obtained by Nessler method to the Michaelis-Menten equation. Studies were performed at 37°C in PBS for hydrolysis of L-Asn. Michaelis-Menten curves for: (**A**) EcAIII, (**B**) KpAIII, (**C**) ReAIV and (**D**) ReAV.

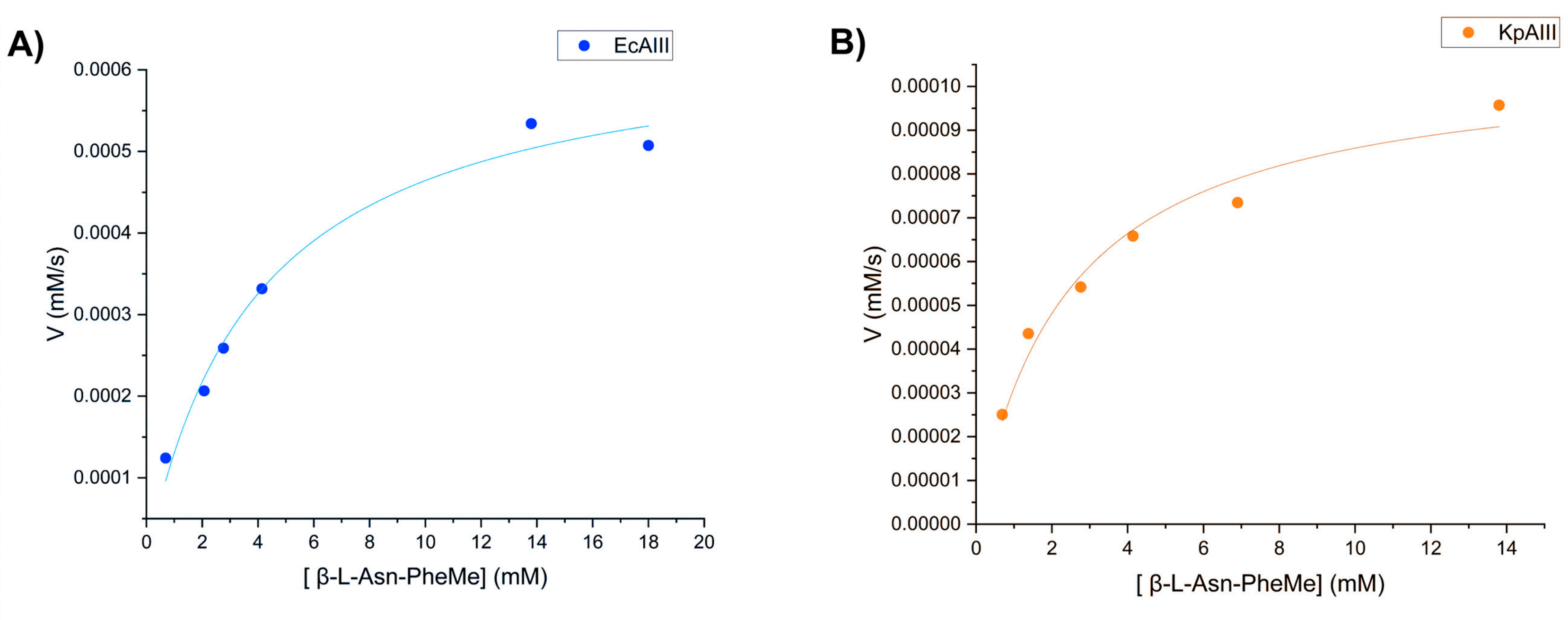

Figure S7. Fitting of kinetic data obtained by GOT method to the Michaelis-Menten equation. Studies performed (37°C, PBS) for hydrolysis of β-L-Asn-PheMe for (**A**) EcAIII and (**B**) KpAIII.
